# Optimization of SAW Devices with LGS/Pt Structure for Sensing Temperature

**DOI:** 10.3390/s20092441

**Published:** 2020-04-25

**Authors:** Xueling Li, Wen Wang, Shuyao Fan, Yining Yin, Yana Jia, Yong Liang, Mengwei Liu

**Affiliations:** 1 Institute of Acoustics, Chinese Academy of Sciences, Beijing 100190, China; lixueling@mail.ioa.ac.cn (X.L.); fanshuyao@mail.ioa.ac.cn (S.F.); yinyining@mail.ioa.ac.cn (Y.Y.); jiayana@mail.ioa.ac.cn (Y.J.); liangyong@mail.ioa.ac.cn (Y.L.); liumw@mail.ioa.ac.cn (M.L.); 2 School of Electronic, Electrical and Communication Engineering, University of Chinese Academy of Sciences, Beijing 100190, China

**Keywords:** surface acoustic wave, Pt/LGS, short pulse method, high-temperature sensor, COM model

## Abstract

Research has shown that SAW (surface acoustic wave) devices with an LGS/Pt (langasite La_3_Ga_5_SiO_14_/platinum) structure are useful in high-temperature sensor applications. Extreme high temperature brings great acoustic attenuation because of the thermal radiation loss, which requires that the sensing device offer a sufficiently high quality factor (Q) and a low loss. Therefore, it is necessary to improve the performance of the quality factor as much as possible so as to better meet the application of high-temperature sensors. Based on these reasons, the main work of this paper was to extract accurate simulation parameters to optimize the Pt/LGS device and obtain Q-value device parameters. Optimization of SAW devices with LGS/Pt structure for sensing extreme high temperature was addressed by employing a typical coupling of modes (COM) model in this work. Using the short pulse method, the reflection coefficient of Pt electrodes on LGS substrate was extracted accurately by characterizing the prepared SAW device with strategic design. Other relevant parameters for COM simulation were determined by finite element analysis. To determine the optimal design parameters, the COM simulation was conducted on the SAW sensing device with a one-port resonator pattern for sensing extreme temperature, which allows for a larger Q-value and low insertion loss. Experimental results validate the theoretical simulation. In addition, the corresponding high-temperature characteristics of the prepared sensing device were investigated.

## 1. Introduction

**Figure 1:**
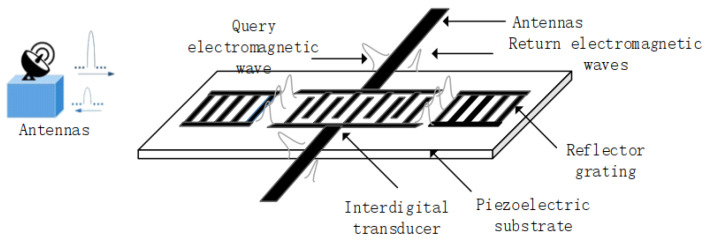
Wireless and passive SAW (surface acoustic wave) sensor systems employing a one-port resonator.

Traditional sensing technology is not an adequate candidate for sensing extreme high temperature because of its wired installation and power supply requirement. Surface acoustic wave (SAW)-based devices provide a perfect way for sensing extreme high temperatures because it features passive operation with no need for a separate power supply, and makes wireless installation at particularly inaccessible locations possible. The typical scheme of a wireless and passive SAW sensor system employing a one-port resonator pattern is depicted in Figure 1. The interdigital transducers (IDTs) receive the electromagnetic wave (EM) energy from the reader unit via the antenna, and a SAW is generated and propagates toward the reflectors. The reflected SAW from the reflectors in each side of the IDTs is reconverted into EM waves by the IDTs and transmitted to the reader unit. The perturbation from the external physical quantities modulates the SAW propagation, inducing a change in resonation frequency. By evaluating the frequency shifts, the corresponding physical quantities can be determined. Obviously, using the heat-resistant piezoelectric substrate and metal electrodes, SAW devices provide a great option for sensors operating at extreme high temperature. As a typical piezoelectric crystal for high-temperature sensors, langasite (La_3_Ga_5_SiO_14_, LGS) attracts widespread attention because of its excellent acoustic properties at high temperature, which exceed 800 °C [1,2,3,4]. Employing LGS and heat-resistant metal electrodes as platinum (Pt) with large melting point, LGS-based SAW devices were reported frequently for sensing strain, pressure, and even gases at extreme high temperature [5,6,7,8,9,10,11]. Al_2_O_3_ was always chosen as a protective layer to improve the stability of LGS/Pt structure. Canabal et al. proposed and successfully realized a prototype of a LGS-based SAW sensor for wireless temperature sensing up to 900 °C [12]. In addition, the LGS device was also explored for pressure sensing at high temperature (over 500 °C), where the maximum pressure value can reach 225 psia [13]. Thierry Aubert et al. offered the temperature characteristics of an LGS-based device with various Euler angles of (0°, 138.5°, 26.7°) and (0°, 22°, 31°) at temperatures up to 700 °C. The influence from Pt electrode thickness on the temperature coefficient of frequency (TCF) of the corresponding devices was addressed [7].

Obviously, the quality factor (Q) and acoustic attenuation of the SAW device will be crucial for the wireless transmission performance [14]. Extreme high temperature will bring great acoustic attenuation because of the thermal radiation loss, which requires the sensing device to offer a sufficiently high Q and lower loss. The high-temperature sensing mechanism and production process were the focus of attention in previous research, but the optimal design of the device pattern allowing for high Q-value and low loss was not thoroughly investigated. In this paper, a high-temperature experiment was conducted. 

Actually, the premise of realizing a high-performance sensing device is to establish an accurate optimization simulation method. The typical and fast simulation approach for SAW devices is the coupling of modes (COM) model, but the key to achieve accurate simulation lies in the extraction of accurate simulation parameters. Of all COM parameters, the reflection coefficient (*RC*) plays a very important role; however, it is hard to extract accurately. Some approaches were proposed in previous work to obtain the *RC* of the metal grating on top of the piezoelectric crystal. D. A. Simons treated the mechanical load induced by the metal electrodes by using perturbation theory under the condition of the crystal isotropy hypothesis, and the expression of the *RC* of a single electrode was deduced, but the calculated phase of *RC* deviated greatly from the experimental results [15]. S. Datta and B. J. Hunsinger demonstrated the piezoelectric short-circuit effect in *RC* by using the orthogonal mode analysis method. The contribution of mass load and stress load effect in the electrode reflection characteristics was described [16,17]. The theoretical result was more accurate and close to the experimental results. Using the first-order perturbation theory and scattering matrix method, the contribution of mechanical load and electrical load in the *RC* was presented [18]; however, the experimental value was still inconsistent with the theoretical analysis because the phase relationship among them was ignored. 

In this work, the optimization of a device with Pt/LGS structure for sensing extreme high temperature was conducted by employing the typical COM model. The short pulse method was established to accurately determine the *RC* of Pt/Ti electrodes on LGS substrate. On this basis, COM simulation of the high-temperature sensing device with Pt/LGS structure was performed to determine the optimal design parameters, allowing for a high Q-value and low insertion loss. Experimental results from the prepared device with a one-port resonator pattern confirmed the theoretical analysis.

## 2. Extraction of COM Parameters

### 2.1. Reflection Coefficient

Usually, the *RC* of electrodes in SAW device can be defined by [19]:

*RC* = *R_e_* + *R_m_* × *h*/*λ*, (1)

where *R_e_* and *R_m_* denotes the contribution from the piezoelectric short circuit and mechanical load effect, which are constants relating to the materials of piezoelectric substrate and electrodes. The *h*/*λ* indicates the normalized thickness of the electrodes. That means the *RC* of the Pt electrodes on LGS substrate was considered as a constant as long as the normalized electrode thickness remained the same. 

**Figure 2:**
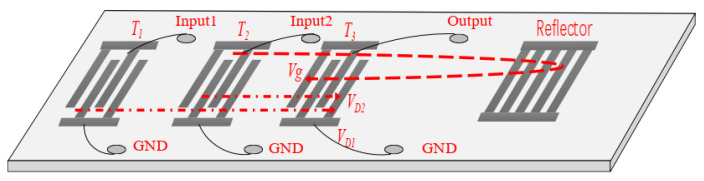
The structure of the SAW device for extraction of reflection coefficient.

The determination of *RC* by the short pulse method is to measure the acoustic reflection characteristics of a SAW device with specific delay line pattern depicted in Figure 2. The SAW device used was composed of three Pt IDTs and one shorted grating Pt reflector on LGS (0°, 138.5°, 26.7°). In order from left to right, they are defined by the transmitting IDT *T_1_*, *T_2_* and the receiving IDT *T_3_*. The length of all the IDTs was set to 15λ, while the reflector includes 100 electrodes. Corresponding parameters for the SAW device are listed in Table 1. The same electrode width of 1/4λ in IDTs and reflectors was designed, and the metallization ratio was set to 0.5.

**Figure 3:**
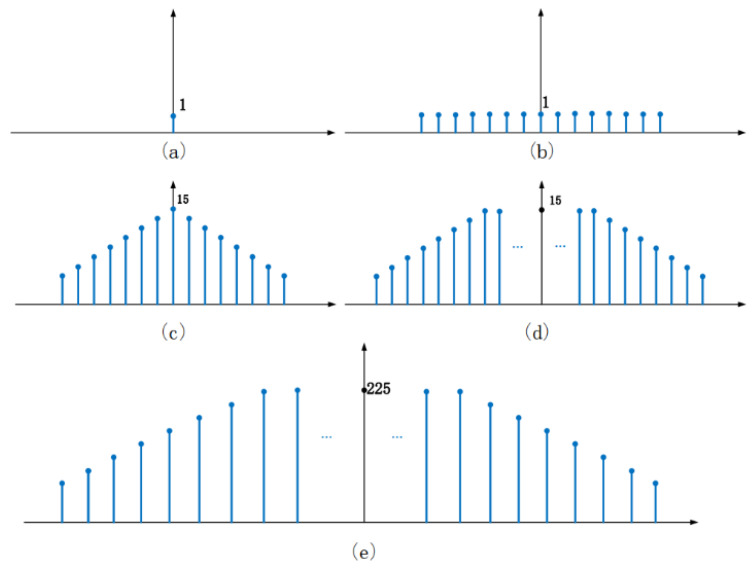
Principle of calculating the reflection coefficient schematic diagram. (**a**). Periodic pulse signal is applied to *T_2_*. (**b**). The excitation pulse signal is convoluted with a 15 cycle pulse sequence. (**c**). The electrical signal converted from SAW propagates to *T*_3_. (**d**). The convolution of the incident SAW and the transfer function of the reflectors. (**e**). Reflection signal *V_g_* from *T*_3_.

The calculation principle of *RC* is described as follows. As shown in Figure 2, *T_3_* receives signal *V_D2_* generated by *T*_2_ and reflection signal *V_g_* from reflector. The *RC* denoted by *r* can be determined by analyzing *V_D_*_2_ and *V_g_*. The reflected acoustic wave (*V_g_*) propagates through *T*_3_ once more than the signal *V_D_*_2_ generated by *T*_2_, so part of the energy is attenuated, and the acoustic wave attenuation occurs accordingly. *T*_1_ is used to determine the attenuation factor denoted by *γ*. When one or more electrical pulse signals are applied for *T*_2_, they will be converted into SAW owing to the piezoelectric effect. SAW propagates along the piezoelectric substrate and reaches *T*_3_, and a considerable part of the acoustic wave energy can be reconverted into electrical signals. But the left SAW continues to propagate and is reflected after reaching the reflector. The reflected SAW propagates through *T*_3_, and the corresponding reflected signal *V_g_* can be obtained. Then, the *RC* (*r*) can be extracted by comparing the maximum amplitude of *V_D_*_2_ with the electrical signal *V_g_*. To facilitate the understanding of the above-mentioned signal propagation and transformation mechanism, a schematic diagram was introduced as depicted in Figure 3. The signal is represented by a sequence. The vertical axis denotes the signal strength and is dimensionless, and the horizontal axis shows the amplitude. As shown in Figure 3a, the amplitude of a periodic pulse signal is assumed to be 1, and applied to *T*_2_. The transfer function of the pulse sequence amplitude of *T*_2_ includes a 15 cycle pulse sequence with an amplitude of 1. The excitation pulse signal is convoluted with a 15 cycle pulse sequence, and the resulting signal is a 15 cycle pulse sequence as depicted in Figure 3b. The corresponding amplitudes are arranged in chronological order as (1 1 1 1 1 1 1 1 1 1 1 1 1 1 1 1 1 1 1 1 1 1 1 1 1 1 1 1 1 1 1 1 1 1 1 1 1). The SAW propagates to *T*_3_ and part of the energy is converted into the electrical signal, as shown in Figure 3c, which is expressed by a 15 cycle pulse sequence, and their amplitude is also arranged in chronological order as (1 2 3 4 5 6 7 8 9 10 11 12 14 14 11 11 10 9 8 5 4 3 2 1). The transmitted SAW is reflected when it reaches the reflector. The reflected signal shown in Figure 3d is the convolution of the incident SAW and the transfer function of the reflectors. The pulse length after convolution is 114, and the amplitude is set in time order as (1 2 3 4 5 6 7 8 9 10 11 12 14 15 15…15 15 14 13 11 11 10 8 7 6 5 4 2 1). When the SAW signal reaches the receiving transducer *T*_3_, the signal is reconvolved with the *T*_3_ transfer function to obtain the 128 cycle impact signal, as shown in Figure 3e, the amplitude of them is set in chronological order as (1 3 6 10 15 21 28 36 45 55 66 78 91 105 120 134 147 159 170 180 189 197 204 210 215 219 222 224 225…225 225 224 222 219 210 204 197 189 170 159 147 134 120 105 91 78 66 55 45 36 28 21 15 10 6 3 1).

It is assumed that *V_0_* is the response amplitude of an IDT excited by a periodic pulse. Hence, the maximum response amplitude *V_D_*_2_ can be expressed by

*V_D_*_2_ = 15*V*_0_ (2)

while the maximum amplitude of the reflected signal *V_g_* was defined by

*V_g_* = *225rV*_0_ (3)

Therefore,

*V_0_* = *V_D_*_2_/15 = *V_g_*/225*r* (4)

Then, the reflection attenuation *r* can be expressed as

*r* = 15*V_g_*/225*V_D_*_2_ = *V_g_*/15*V_D_*_2_ (5)

Considering that the attenuation *γ* of the SAW propagates to the receiving transducer in the first time, Equation (4) should be changed to

r = *V_g_*/15 *ƞV_D_*_2_ (6)

where

ƞ = *V_D_*_1_/*V_D_*_2_ (7)

**Figure 4:**
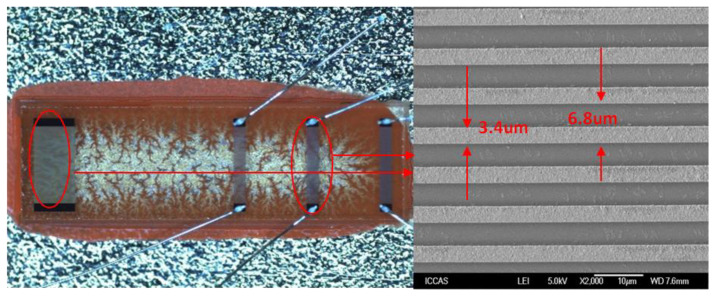
The physical picture of sensor chip that was developed.

Next, the *RC* of the Pt electrodes on LGS was extracted experimentally by characterizing the SAW device with a strategic delay line pattern as described in Figure 2. Using the design parameters listed in Table 1, the SAW device, with three photolithographically defined Pt IDTs and one Pt reflector was fabricated on an LGS wafer. The liftoff technique was employed for the Pt electrodes deposition, and to improve the Pt adhesion, a Ti thin film (50 nm) was coated onto the LGS prior to Pt deposition. The ratio of Pt film thickness to wavelength ($h_{pt}/\lambda$) was set about 1.5%. The corresponding device frequency was set to operate at 195 MHz, and the width of electrodes in the IDTs and reflector was 3.4 µm. An optical picture of the prepared SAW device, which was composed of three IDTs and one shortened grating reflector, is depicted in Figure 4.

**Figure 5:**
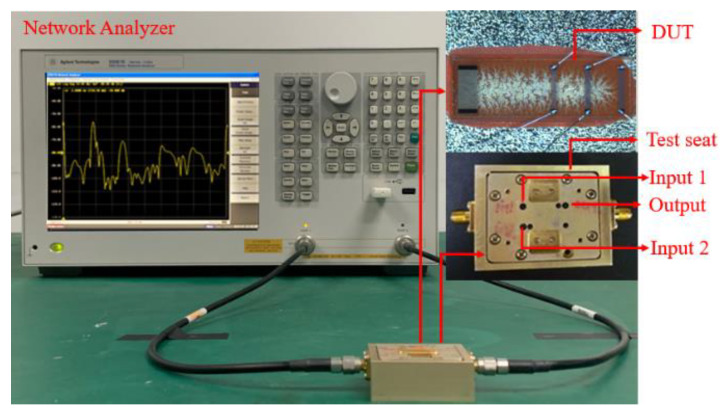
The experimental setup for characterizing the proposed SAW sensing devices.

The *RC* was measured using a network analyzer (Agilent E5061B), as shown in Figure 5. The pulse signal excitation was conducted by the time domain function of the network analyzer. The response waveform of the proposed device in frequency domain and time domain was measured and *RC* was then evaluated by using Equations (6) and (7).

**Figure 6:**
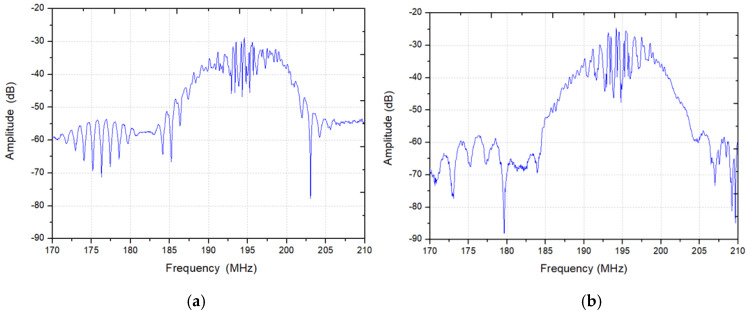
The measured frequency signals from *T*_1_ (**a**) and *T*_2_ (**b**).

**Figure 7:**
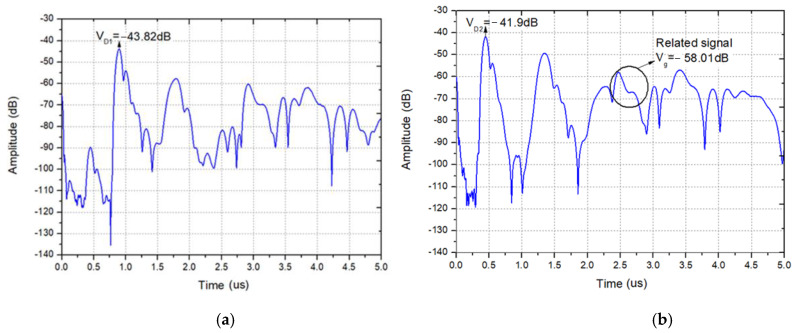
The pulse responses in time domain from *T_2_* to *T_3_* (**a**) and *T_1_* to *T_3_* (**b**).

**Figure 8:**
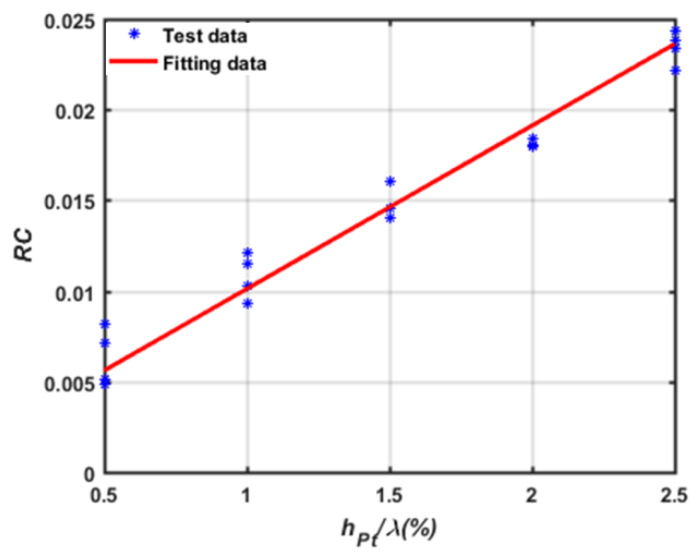
RC of the prepared SAW device with various *h_pt_*/*λ*.

Figure 6 indicates the measured frequency signals from *T*_1_ and *T*_2_. Many ripples in frequency signals as shown in Figure 6 were caused by the multiple reflections from the adjacent reflectors. Corresponding pulse responses in time domain from *T*_2_ to *T*_3_ and *T*_1_ to *T*_3_ are described in Figure 7a and Figure 8b. The maximum amplitude of ~−30 dB in frequency signals occurs at a frequency of ~195 MHz, which was consistent with the design prediction.

There is a pulse signal at the zero point of the time axis in Figure 7, which is an electrical signal directly coupled from the input to the output without acoustoelectric conversion. Due to the fast propagation speed of the electrical signal, the corresponding position in time domain is close to the zero point. The highest pulses in Figure 7a,b appear near 0.9 and 0.4 μs, respectively, which denote the delay line response signal. The following wide signals with lower amplitude are the reflection signal from the adjacent reflectors. In addition, the front end of the reflection signal is higher, and the back section is lower. The reason is that there are multiple acoustic reflections between the gratings, and the partial acoustic energy is reflected after the acoustic wave passes through a grating. The pulses that appear near 1.4 and 1.7 μs in the figure are the three stroke signal of the delay line response signal. *V_D_*_1_ in Figure 7a denotes the delayed response signal from *T*_1_ to *T*_3_, and corresponding delay time and amplitude are ~0.9 µs and −43.82 dB. Due to the acoustic wave propagation attenuation, the amplitude of the reflection signal decreases with the increasing propagation distance. Figure 7b gives the impulse response waveforms in time domain from *T_2_* to *T_3_*. The response amplitude *V_D_*_2_ is evaluated as −41.9 dB, marked by an arrow in Figure 7b. Using Equations (2)–(6), the attenuation coefficient γ obtained is calculated as 0.8017. In addition, the maximum amplitude of reflection signal *V_g_* is observed as −58.01 dB, marked by the circle and arrow in Figure 7b, hence, the *RC* is deduced as 0.0133 by using the expression described in Equations (2)–(5). 

Then, the contributions to the reflection coefficient from the piezoelectric short circuit and mechanical load effect of the Pt electrodes were determined by extracting the RC from the Pt electrode with various thicknesses according to Equation (7). The normalized Pt thicknesses were set to 0.5–2.5%. Employing this approach, the RC values of the Pt electrode with various normalized thicknesses were extracted, as shown in Figure 8. Obviously, the RC value increases linearly with the normalized Pt thickness, and then, the Re and Rm in Equation (7) can be determined by linear fitting, as shown in Equation (8).

(8) $$RC=3.3\times 10^{-4}+0.0082\times h_{pt}/\lambda$$

### 2.2. Extraction of Other Relevant Parameters

With the exception of the reflection coefficient, other relevant parameters for COM simulation, i.e., propagation velocity *v*, coupling coefficient *κ*, excitation coefficient *α*, and static capacitance C, as defined by Equations (9)–(11), were extracted by the three-dimensional finite element method (FEM) method.

(9) $$\{\begin{array}{l} v=\lambda \frac{\left(f_{\mathrm{sc}+}+f_{\mathrm{sc}-}\right)}{2},\\ \left|\kappa \right|=\frac{2\pi }{\lambda }\frac{f_{\mathrm{sc}+}-f_{\mathrm{sc}-}}{f_{\mathrm{sc}+}+f_{\mathrm{sc}-}}. \end{array}$$

(10) $$\{\begin{array}{l} \left|\alpha \right|=\sqrt{\frac{\omega C_{n}W\pi }{\lambda^{2}}\left(\frac{f_{\mathrm{oc}+}+f_{\mathrm{oc}-}}{f_{\mathrm{sc}+}+f_{\mathrm{sc}-}}-1\right)},\\ \cos\left(∠\alpha^{2}/\kappa \right)=\frac{{\left(f_{\mathrm{oc}+}-f_{\mathrm{oc}-}\right)}^{2}-{\left(f_{\mathrm{sc}+}-f_{\mathrm{sc}-}\right)}^{2}-{\left[\left(f_{\mathrm{oc}+}+f_{\mathrm{oc}-}\right)-\left(f_{\mathrm{sc}+}+f_{\mathrm{sc}-}\right)\right]}^{2}}{2\left(f_{\mathrm{sc}+}-f_{\mathrm{sc}-}\right)\left[\left(f_{\mathrm{oc}+}+f_{\mathrm{oc}-}\right)-\left(f_{\mathrm{sc}+}+f_{\mathrm{sc}-}\right)\right]}. \end{array}$$

(11) $$C_{n}=\frac{W_{e}}{{\left(\Delta V\right)}^{2}W}.$$

**Figure 9:**
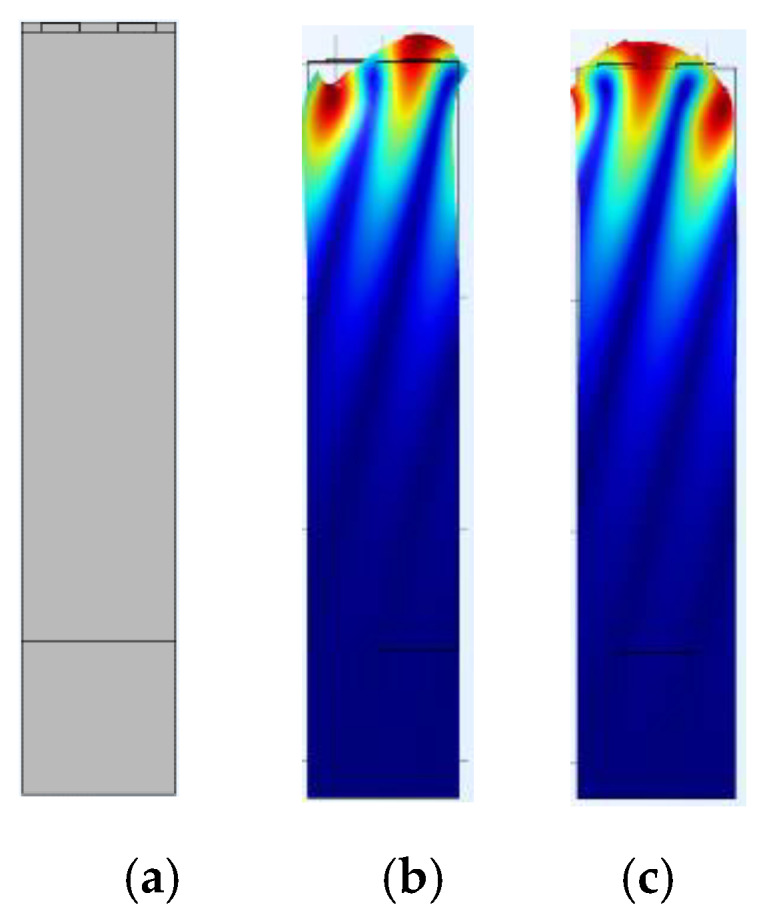
Finite element model (FEM) analysis applied to the acoustic modes in Pt/LGS: (**a**) device structure in FEM, (**b**) symmetric model, and (**c**) antisymmetric model.

**Figure 10:**
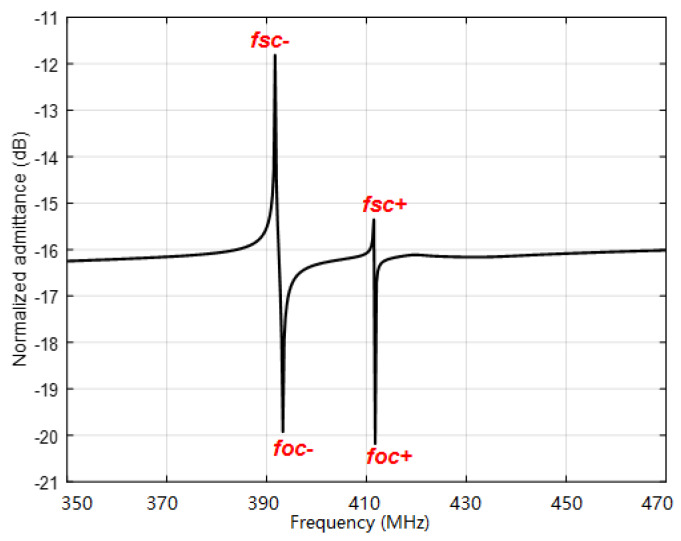
Normalized input admittance.

Here, *f_sc+_*, *f_sc-_*, *f_oc+_*, and *f_oc-_* denote the up and down boundary frequency of the stopband in a periodic shortened grating and open grating. *W* and *λ* are the acoustic aperture and corresponding wavelength. Using the FEM method (COMSOL 5.3 Multiphysics software) and the mechanical parameters of the LGS piezoelectric substrate and Pt electrodes [20], the modal analysis towards SAW propagations in Pt IDTs/LGS substrate can be effectively conducted, and the corresponding SAW displacement profile in IDTs calculated as depicted in Figure 9. Following the modal analysis, the harmonic response analysis was performed to achieve the admittance characteristics, and the corresponding *f_sc+_*, *f_sc-_*, *f_oc+_*, and *f_oc-_* extracted by searching the eigenfrequencies in normalized admittances, as shown in Figure 10, and allowing the extraction of COM parameters as SAW velocity, coupling coefficient, and excitation coefficient. Moreover, a static analysis was performed to the structure of the IDTs/piezoelectric substrate to determine the static capacitance. Table 2 summarizes the extracted parameters for COM simulation.

## 3. COM Model

**Figure 11:**
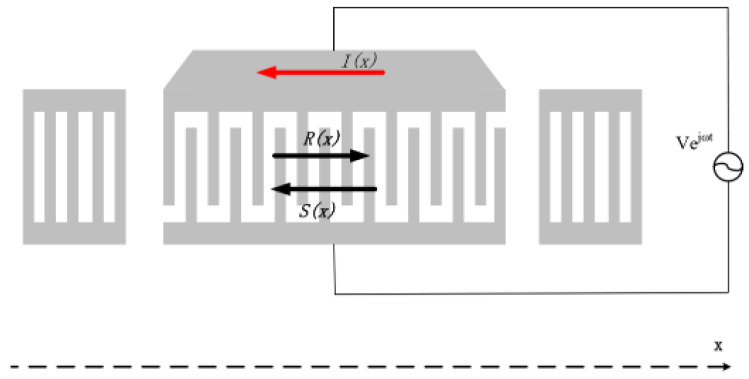
The COM model of the sensing device patterned by a one-port resonator.

The COM model attracts great attention because simulation is a fast and simple way to determine the optimal design parameters of the SAW device prior to fabrication. However, as an approximate phenomenological model, the accuracy in the simulation was decided by the COM parameters. Using the extracted COM parameters given in Section 2, the high-temperature sensing device patterned by a one-port resonator (Figure 11) with the structure of Pt/LGS can be simulated, and corresponding optimal design parameters were determined.

For simulation of the one-port resonator configuration, composed of one IDT and two adjacent shortened grating reflectors (Figure 11), the COM model was used to analyze the IDT and reflectors reflectively. By using the cascading mixed P-matrix of the IDT, reflectors, and cavity between the IDT and adjacent reflectors, the frequency response *S*_11_ can be expressed by

(12) $$S_{11}=20\times \log((R\times P_{33}-1)/(R\times P_{33}+1)),$$

where *R* denotes the match impedance of the input and output port. In addition, the real part of the cascading *P*-matrix element *P*_33_ represents the conductance, indicating the *Q*-value of the devices.

**Figure 12:**
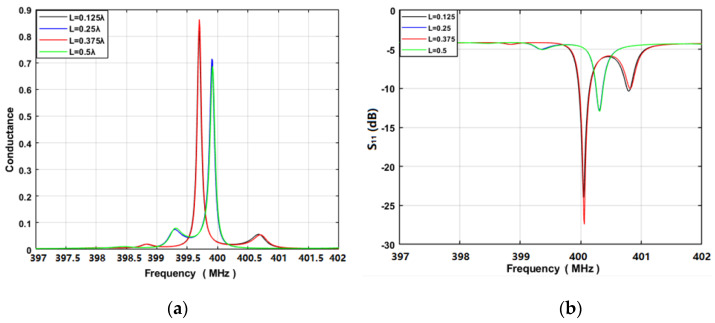
COM simulation on sensing device: (**a**) conductance, (**b**) S_11_.

Next, the performance of the sensing device with the structure of Pt/LGS was predicted by using the COM model described previously, and the corresponding optimal design parameters were determined by varying the structure parameters listed in Table 3. The device was set to operate at 400 MHz. The corresponding Pt electrode width was 1.57 µm and the Pt thickness was set to 100 nm. Figure 12 shows the simulated S_11_ and conductance of the proposed high-temperature sensing device. Obviously, a significant effect appeared from the cavity between the IDT and the adjacent reflectors, which lies in the phase difference of the acoustic wave in the resonance cavity. Obviously, the cavity of 0.375λ allows a high Q-value.

## 4. Experimental Results

**Figure 13:**
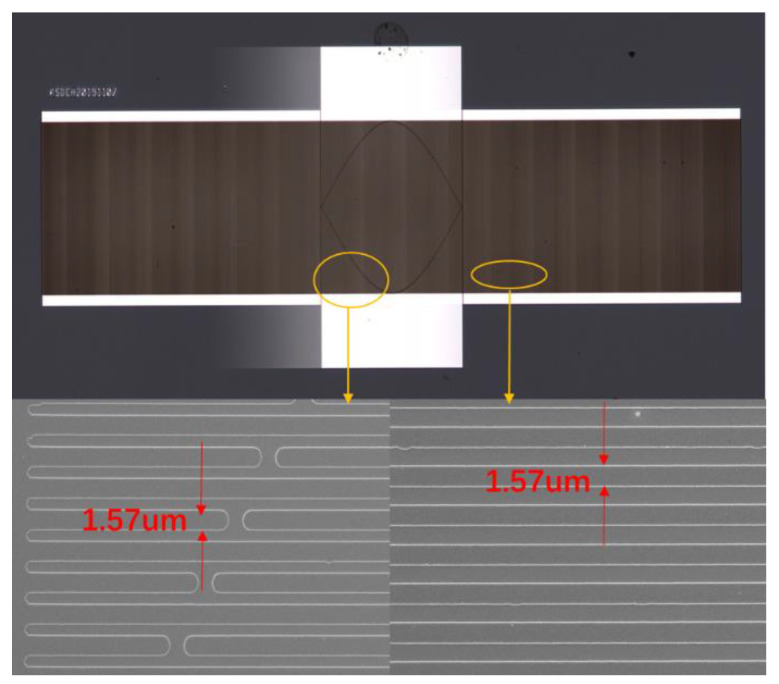
Optical picture of the proposed sensing devices.

Based on the COM simulation, the sensing chip for the high-temperature sensing device was reproducibly prepared on a LGS (0°, 138.5°, 26.7°) substrate by the standard photolithographic technique. The cavity of 0.375λ between the IDT and reflectors was chosen, and other structure parameters were similar to the parameters listed in Table 3. The fabrication procedure of the sensing device is depicted in Figure 13. Ninety-five nm of Pt (normalized thickness of 1.5%) was deposited on the cleaned LGS substrate surface using a thermal evaporate. Prior to Pt deposition, a Ti thin film was coated onto the substrate to improve the adhesive of the Pt on LGS. Then, a 1-mm-thick photoresist (PR) was spin-coated, exposed, and developed for the resonator pattern. The Pt/Ti was stripped and the PR was dissolved in acetone. Finally, the piezoelectric wafer with the SAW device pattern was dicing-sawed for wafer bonding and packaging. Figure 13 shows the optical picture of the proposed sensing devices; the width of Pt electrodes was 1.57 µm. 

**Figure 14:**
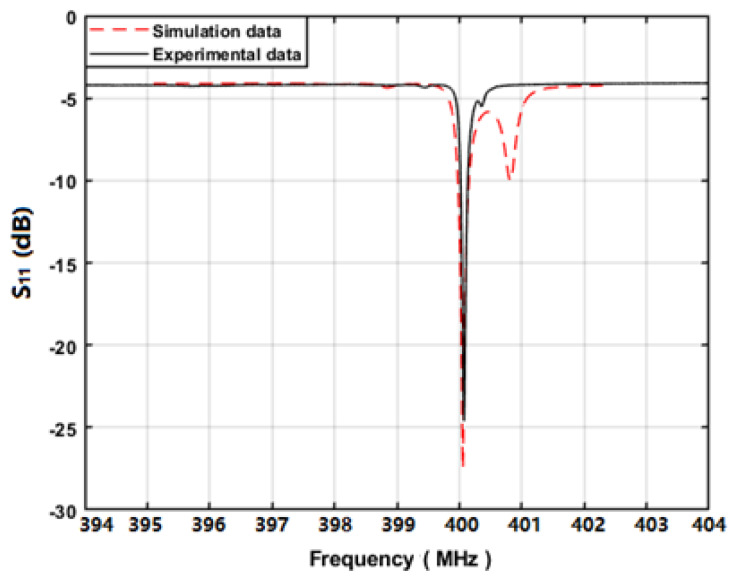
The measured S_11_ of the prepared SAW sensing devices.

Using the network analyzer, the prepared sensing device with a cavity of 0.375λ was characterized, as shown in Figure 14. The measured operation frequency of the proposed device was ~400 MHz, agreeing well with the design parameters. The obtained unloaded Q-value was up to 4970 from the device with cavity of 0.375λ. In addition, the measured S_11_ was well matched with the predicted values from the COM simulation. The structure of LGS/Pt generated both Rayleigh wave and weak shear wave mode. The spurious response that occurred at 401 MHz indicates the shear wave mode. Additionally, the spurious response in the measurement was more obvious compared to the theoretical simulation. One possible reason was that the Ti adhesive layer in COM simulation was neglected. Overall, the measured result fully proves the accuracy of the extracted COM parameters, especially the reflection coefficient. 

**Figure 15:**
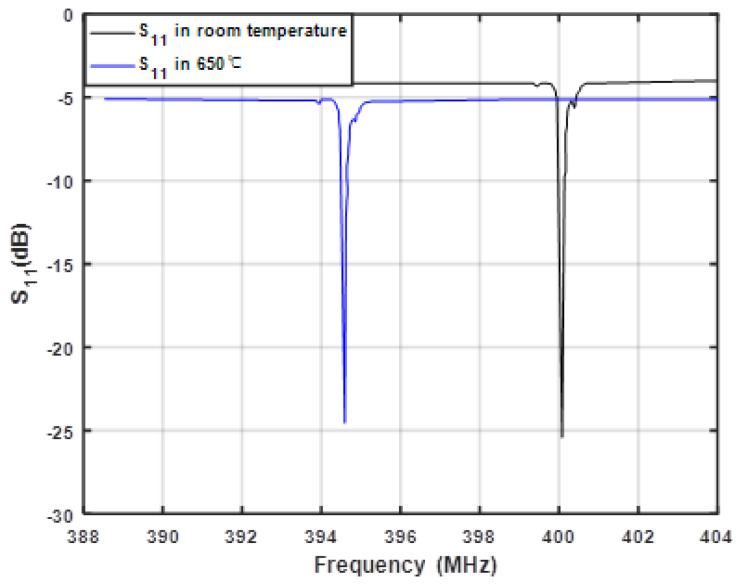
The measured S_11_ of the prepared SAW sensing devices at various temperatures.

**Figure 16:**
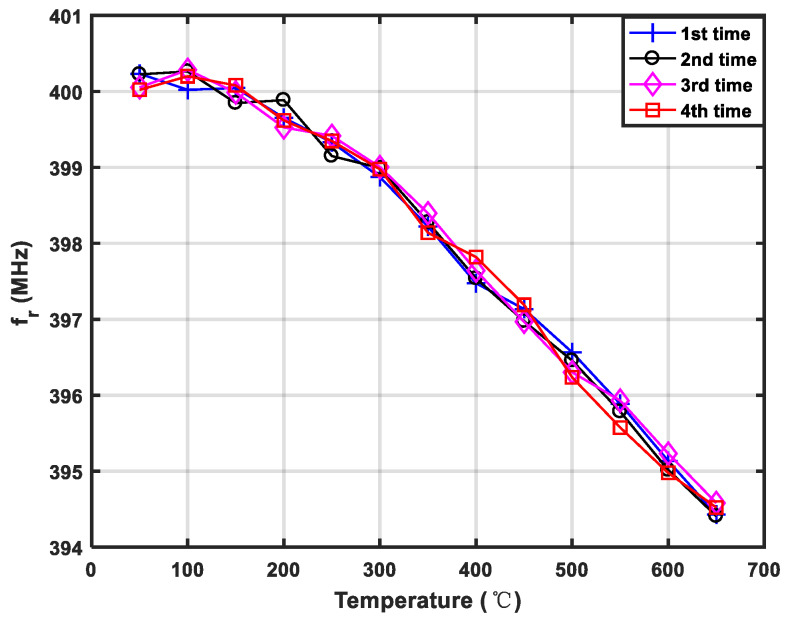
The measured frequency shift according to temperature (50–650 °C).

Next, the measured S_11_ at room temperature and 650 °C are shown in Figure 15. Obviously, after the temperature changes, the frequency of the device drifted by 5.5 MHz, but the return loss and the quality factor did not change significantly, which shows that the performance of the sensor device with Pt/LGS structure was relatively stable in the high-temperature environment. The corresponding shifts in frequency relating to the changed temperature are described in Figure 16. Here, the prepared SAW devices were tested in the temperature range of 50–650 °C at the interval of 50 °C. Approximately linear frequency shifts according to temperature (50–650 °C) were observed, and the corresponding temperature coefficient of frequency (Tcf) was evaluated as ~25 ppm/°C. 

## 5. Conclusions

A high-temperature environment will mainly lead to obvious acoustic attenuation, which will sharply reduce the quality factor of the sensing device. Therefore, it is necessary to improve the performance of the quality factor as much as possible. In this work, an accurate reflection coefficient to optimize the Pt/LGS device was extracted by using the short pulse method. Based on the structure of the LGS/Pt device with a different ratio of Pt film thickness to wavelength, the experiment was carried out. The least squares method was used to fit the experimental data, and the calculation formula of the reflection coefficient was characterized. The other COM parameters of Pt/LGS were extracted by FEM approaches. Using the typical COM model, the one-port resonator patterned device with the structure of Pt/LGS was simulated, and the optimal design parameters allowing a larger Q-value were determined. Based on the simulation, a 400 MHz one-port SAW resonator with Pt electrodes on LGS substrate was photolithographically developed, and then characterized by using a network analyzer. A high Q-value was obtained, and the measured result agreed well with the simulation. In addition, the high-temperature characteristics of the prepared sensing device were investigated at a temperature of 50–650 °C; excellent stability and linear TCF were observed, which indicate the prepared sensing device was promising for high-temperature sensing. 

## Figures and Tables

**Table 1:** Design parameters for the SAW device.

Parameters	Description	Values
*λ*	Wavelength	13.6 µm
*E*	Electrode width	3.4 µm
*A*	Aperture	1.36 mm
*f*	Frequency	195 MHz
*N_int_*	Number of *T*_1_ and *T*_2_ pairs	15
*L*	Distance between IDTs	1 mm
*L_1_*	Distance between Reflector and *T*_3_	2.6 mm

**Table 2:** COM parameters of SAW device with structure of Pt/LGS.

COM Parameters	*v* (m/s)	α	C (F)	*κ*
value	2512	1.613 × 10^−4^	2.589 × 10^−10^	0.0133

**Table 3:** One-port SAW resonator design parameters.

λ (µm)	Number of IDT Pairs	Number of Reflector Pairs	W	L1	L2
6.28	60	180	100λ	0.125λ	0.125λ
6.28	60	180	100λ	0. 25λ	0.25λ
6.28	60	180	100λ	0.375λ	0.375λ
6.28	60	180	100λ	0.5λ	0.5λ

## References

[1] Fukuda T., Takeda P., Shimamura K., Kawanaka H., Kumatoriya M., Murakami S., Sato J., Sato M. Growth of new langasite single crystals for piezoelectric applications. Proceedings of the Eleventh IEEE International Symposium on Applications of Ferroelectrics.

[2] Chai B., Lefaucheur J.L., Ji Y.Y., Qiu H. Growth and evaluation of large size LGS, LGN and LGT single crystals. Proceedings of the IEEE International Frequency Control Symposium.

[3] Buzanov O.A., Naumov A.V., Nechaev V.V., Knyazev S.N. A new approach to the growth of langasite crystals. Proceedings of the IEEE International Frequency Control Symposium.

[4] Smythe R.C. Material and resonator properties of langasite and langatate—A progress report. Proceedings of the IEEE International Frequency Control Symposium.

[5] Li L., Peng B. (2019). Temperature-Dependent Characteristics of Surface Acoustic Wave Resonators Deposited on (0°, 138.5°, ψ) Langasite Cuts. IEEE Sens. J..

[6] Ayes A., Bernhardt G., da Cunha M.P. Removal of Stress Hillocks from Platinum-Alumina Electrodes Used in High-temperature SAW Devices. Proceedings of the IEEE Ultrasonics Symposium.

[7] Aubert T., Nicolay P., Sarry F. Thermoelastic effects in Pt IDTs. Impact on the behavior of high-temperature LGS-based SAW devices. Proceedings of the IEEE International Ultrasonics Symposium (IUS).

[8] Weihnacht M., Sotnikov A., Schmidt H., Wall B., Grünwald R. Langasite: High temperature properties and SAW simulations. Proceedings of the International Ultrasonics Symposium.

[9] Shu L., Peng B., Yang Z., Wang R., Deng S., Liu X. (2015). High-Temperature SAW Wireless Strain Sensor with Langasite. IEEE Sens. J..

[10] Bardong J., Aubert T., Naumenko N., Bruckner G., Salzmann S., Reindl L.M. (2013). Experimental and Theoretical Investigations of Some Useful Langasite Cuts for High-Temperature SAW Applications. IEEE Trans. Ultrason. Ferroelectr. Freq. Control.

[11] Sakharov S., Kondratiev S., Zabelin A., Naumenko N., Azarov A., Zhgoon S., Shvetsov A. Theoretical and experimental investigation of langasite as material for wireless high temperature SAW sensors. Proceedings of the IEEE International Ultrasonics Symposium.

[12] Canabal A., Davulis P.M., Pollard T., Da Cunha M.P. Multi-Sensor Wireless Interrogation of SAW Resonators at High Temperatures. Proceedings of the 2010 IEEE International Ultrasonics Symposium.

[13] Moulzolf S.C., Behanan R., Lad R.J., da Cunha M.P. Langasite SAW Pressure Sensor for Harsh Environments. Proceedings of the 2012 IEEE International Ultrasonics Symposium.

[14] Wendt T.M., Reindl L.M. Multiple Access Methods utilized to extend Operational Life Time of Wireless Sensor Nodes. Proceedings of the 2008 2nd Annual IEEE Systems Conference.

[15] Simons D.A. (1978). Reflection of Rayleigh Waves by strips, grooves, and periodic arrays of strips or grooves. J. Acoust. Soc. Am..

[16] Datta S., Hunsinger B.J. (1979). First-order reflection coefficient of surface acoustic waves from thin strips overlays. J. Appl. Phys..

[17] Datta S., Hunsinger B.J. (1980). An analytical theory for the scattering of surface acoustic waves by a single electrode in a periodic array on a piezoelectric substrate. J. Appl. Phys..

[18] Skeie H. (1970). Electrical and Mechanical Loading of a Piezoelectric Surface supporting surface waves. J. Acoust. Soc. Am..

[19] He S., Chen D., Wang C. (1990). The IDT with high internal reflection suppression. Acta Acust..

[20] Plessky V., Koskela J. (2000). Coupling-of-modes analysis of SAW devices. Int. J. High Speed Electron. Syst..

